# X-ray therapy promotes structural regeneration after spinal cord injury in a rat model

**DOI:** 10.1186/s13018-015-0327-0

**Published:** 2016-01-13

**Authors:** Dong Liu, Jun Hua, Qi-rong Dong, Yong-ming Sun, Min-feng Gan, Yi-xin Shen, Zhi-hai Fan, Peng Zhang

**Affiliations:** Department of Orthopaedics, the Second Affiliated Hospital of Soochow University, Suzhou, 215004 China; Department of Orthopaedics, the Seventh People’s Hospital of Suzhou, Suzhou, 215151 China; Department of Orthopaedics, the First Affiliated Hospital of Soochow University, Suzhou, 215006 China

**Keywords:** Rat, X-rays, Therapy, Spinal cord injuries

## Abstract

**Objective:**

This study aims to investigate the therapeutic effects and mechanisms of x-ray treatment on rats following spinal cord injury (SCI).

**Methods:**

Forty-six female Sprague–Dawley rats were subjected to spinal cord injury using the modified Allen weight-drop method. The animals were randomly divided into six groups. Two of the animal groups were irradiated with 10 Gy at the lesion site; another two groups were irradiated with 20 Gy; and the last two groups without irradiation were regarded as the sham group. One of the each of two animal groups was euthanized at different time points at 4 and 12 weeks, respectively, after irradiation. Spinal cord calluses were assessed using kinology and electrophysiology and histology methods.

**Results:**

In all of the groups, the neurofilament (NF) counts at 14 weeks were found to be higher than that at 6 weeks after SCI. Both 10-Gy irradiated and 20-Gy irradiated groups were higher than those of the sham group at each time point (*P* < 0.05). The myelin basic protein (MBP) count decreased at 14 weeks after SCI in the irradiated groups (*P* < 0.05) but increased at 14 weeks in the sham group (*P* < 0.05). Furthermore, the MBP count of the irradiated groups was lower than that of the sham group at 14 weeks (*P* < 0.05). The glial fibrillary acidic protein (GFAP) and Nogo-A counts at 14 weeks were higher than those at 6 weeks in all the groups (*P* < 0.05), and there was no statistical significance with kinology and electrophysiology tests in all groups.

**Conclusions:**

A self-repair mechanism exists after spinal cord injury, which lasts at least 14 weeks. X-ray therapy promotes the regeneration of the spinal cord system after injury.

## Introduction

Spinal cord injury (SCI) often leads to progressive spinal cord tissue degeneration and necrosis and then leads to a series of complications involving paralysis, bedsores, infection, and venous embolism. Many therapeutic strategies, such as nerve cell transplantation and hyperbaric oxygen, are currently used, but the results are not satisfactory. In recent years, some scholars have found that a certain dose of x-ray irradiation could promote the recovery of the injured spinal cord in rats, which provided us with a new method of treating spinal cord injuries. However, the dose and time of x-ray irradiation, the animal model of spinal cord injury, and the best observation time points were inconsistent [1–7]. Therefore, x-ray therapy in spinal cord injury needs to be further studied.

The present study investigated the x-ray irradiation treatment in an experimental spinal cord injury model and examined the effect of x-ray irradiation on the recovery of neuromotor functions. Neurofilament (NF) can show nerve fibers clearly, myelin basic protein (MBP) can show the structure of myelin sheaths, and glial fibrillary acidic protein (GFAP) and Nogo-A can show the mesh structure of white matter in the spinal cord. All of these detection indexes can reflect the structure and function of the spinal cord comprehensively. Using counting and statistical analysis, we can realize the regeneration of axons and the microcirculation of the lesion site. Our hypothesis was that x-ray irradiation could change the microcirculation of the injured site, retard the degeneration of the spinal cord, and promote the recovery of structure and function after spinal cord injury in rats.

## Materials and methods

Adult Sprague–Dawley (SD) female rats, 3 to 4 months old, and weighing 235 to 285 g underwent spinal cord injury using the modified Allen weight-drop method [8]. Briefly, rats were injected intraperitoneally with 3.6 % chloral hydrate (1 ml/100 g) under general anesthesia, and the spinal cord T11–12 plane area (approximately 4 × 8 mm) was exposed. A 2.5-mm-diameter metal cylinder weighing 30 g was dropped from a height of 6.0 cm in a vertical plastic tube directly onto the exposed spinal cord, resulting in acute moderate spinal cord injury with an injury force of 180 g·cm. The overlying back muscles and the skin were sutured, and the rats were given an s.c. injection of long-acting penicillin (300,000 units). The bladders were expressed manually two to three times a day until automatic bladder function resumed. The animals were randomly divided into six groups. Two of the animal groups were irradiated with 10 Gy at the lesion site; another two groups were irradiated with 20 Gy; and the last two groups without irradiation were regarded as the sham group. All animals were treated according to the Guide for the Care and Use of Laboratory Animals of the Institute of Laboratory Animal Resources, Commission on Life Science, 1996 National Research Council, Washington DC.

### Radiation

X-irradiation was delivered by a clinical linear accelerator (Siemens) at a dose rate of 200 cGy/min. Treatment was delivered through a posterior approach while the rat was anesthetized at a distance of 100 cm from the skin. The dimensions of the radiation field were 25 × 20 mm (length × width) centered at the site of the lesion. Radiation therapy was delivered as 10 and 20 Gy at 2 weeks postinjury, and selection for treatment was randomized. At this dose level and recovery period until analysis was performed (14 weeks postinjury), no side effects were noticed.

### Neuromotor function assessment

The neuromotor functions of all the rats were examined by the Basso, Beattie, and Bresnahan (BBB) locomotor rating scale [9] before spinal cord injury and 2, 6, and 14 weeks after spinal cord injury. The BBB rating scale has a range from 0 to 21 points, judged by parameters such as the coordination of limb movement, paw placement, and tail balance. No visible movement of the hind legs was scored as 0 point. For the maximum of 21 points, the rat had to walk continuously on the paws, with a cocked tail, good fore and hind limb motor coordination, and truck stability. The toes were required to grip the surface while moving forward, and the paws maintained coordination with the body movement.

### Somatosensory evoked potentials

All of the rats were examined by somatosensory evoked potentials (SEP) before spinal cord injury and 2, 6, and 14 weeks after spinal cord injury. Stimulating electrodes were placed in the tibialis anterior muscles with an intensity of 0.5–1.5 mA and an 1.8-Hz continuous frequency square-wave stimuli. Recording electrodes were placed on the top of the head. The analysis time was 200 ms, bandpass 10~2000 Hz.

### Pathology

Anesthetized rats were perfused with a phosphate-buffered saline solution, and then with a solution of 4 % paraformaldehyde in phosphate-buffered saline, and after removal from the vertebrae, cord tissue samples were further fixed. Fixed cord samples were embedded in paraffin and were sliced into sections, dyed, and examined by light microscopy. Animals in one of each of the two groups which received the same dose of irradiation were euthanized at different time points, namely at 4 and 12 weeks, respectively, after irradiation.

### Statistical analysis

The *t* test and ANOVA for independent samples were performed with the statistical software SAS 8.0. The difference in the comparisons was considered to be statistically significant when *p* was less than 0.05.

## Results

Three rats were eliminated at 1 day and 2 weeks after spinal cord injury by the BBB rating scale and SEP testing, and ten rats died due to postoperative infection or bedsores. There was a mortality rate of 23.3 %. There were nine surviving rats in the sham-operated group, 12 rats in the 10-Gy irradiation group, and 12 rats in the 20-Gy irradiation group. There were no significant differences detected in the BBB rating scale and SEP testing before and after irradiation in each group.

**Fig. 1:**
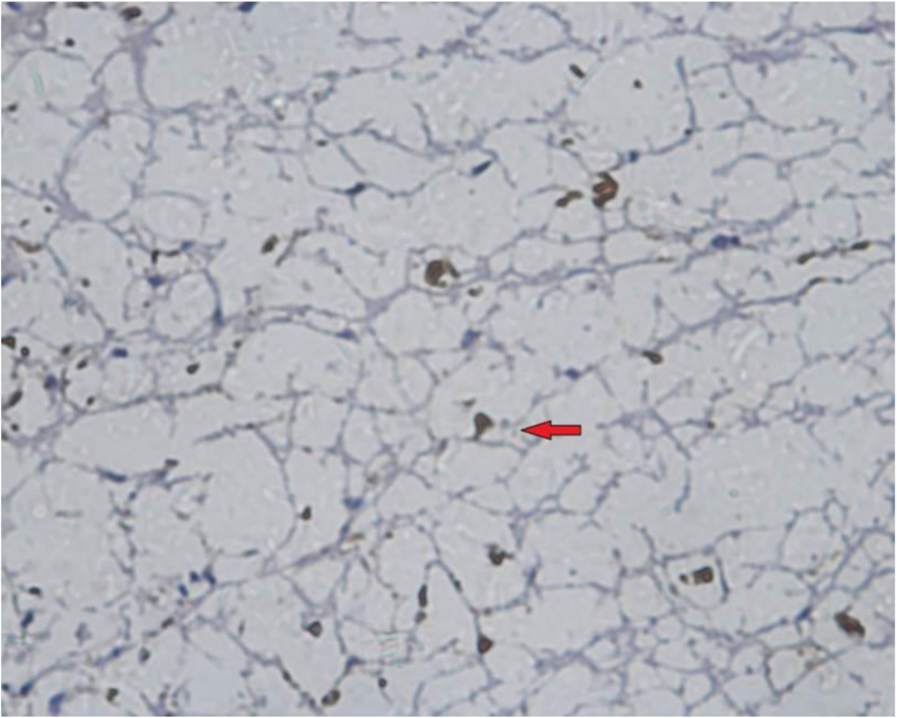
NF sham group 6 weeks after SCI. The myelin staining is light, and part of the grid structure is lost, leaving only the axon in the injury area

**Fig. 2:**
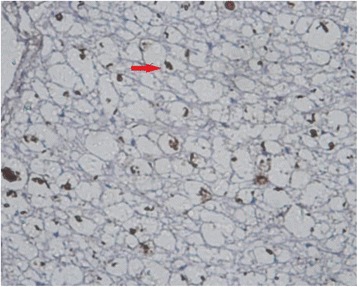
NF sham group 14 weeks after SCI. In the injury area, the myelin staining is light, and the grid structure is little or lost. The axon staining is concentrated

**Fig. 3:**
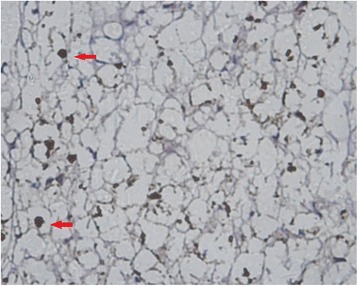
NF 20-Gy group 6 weeks after SCI. In the injury area, the myelin staining is light, and the grid structure is little or lost. The axon staining is concentrated

**Fig. 4:**
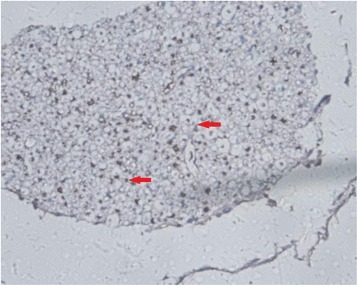
NF 20-Gy group 14 weeks after SCI. The myelin staining is light, and part of the grid structure has ruptured. The axon staining is concentrated

**Fig. 5:**
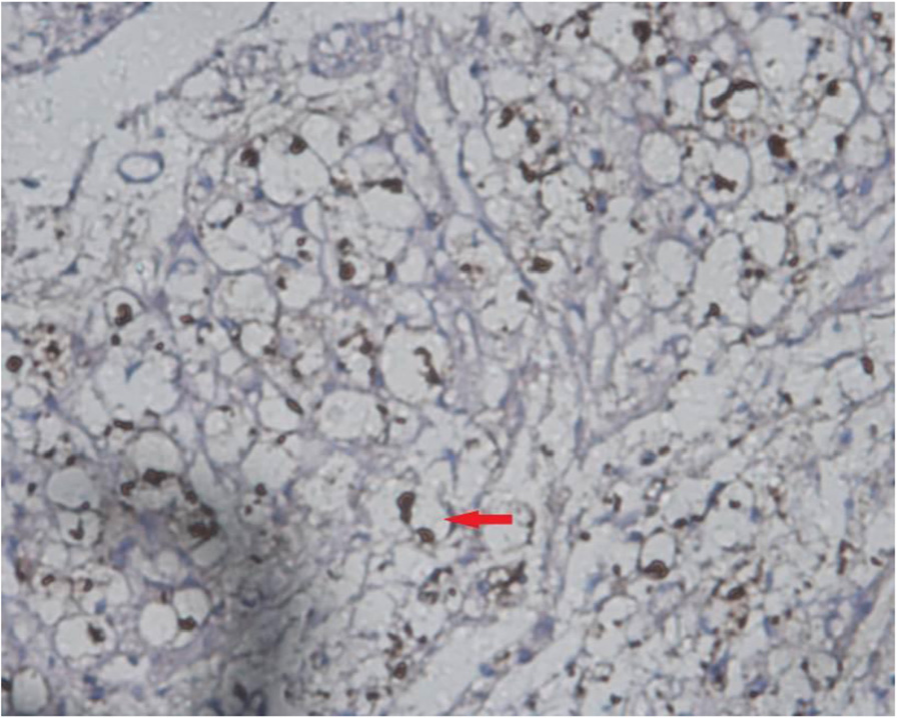
NF 10-Gy group 6 weeks after SCI. The axon is located in the center of the concentric structure, and part of the grid structure has ruptured

**Fig. 6:**
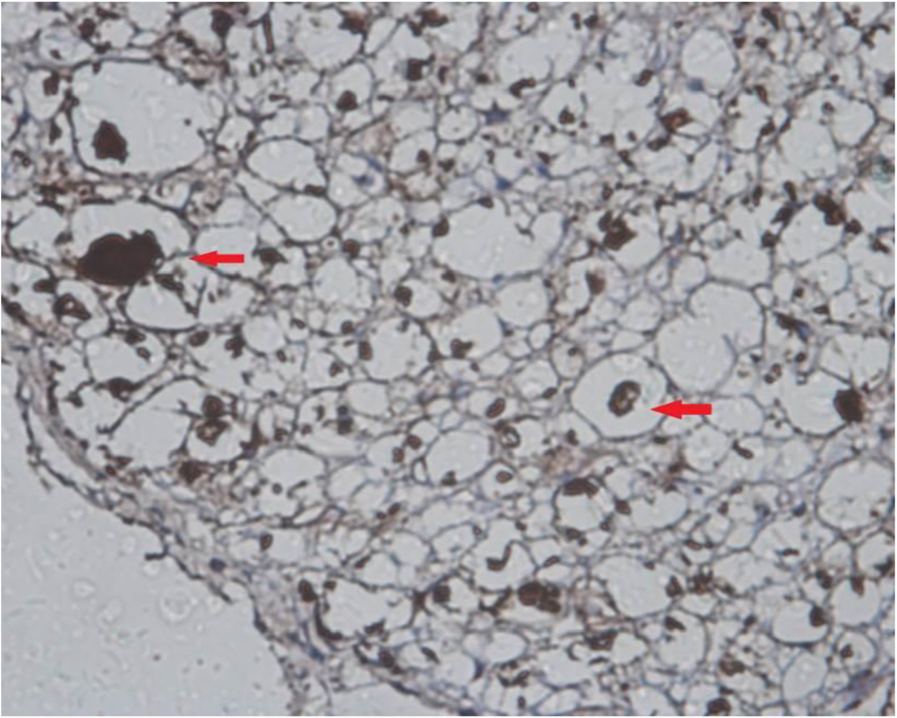
NF 10-Gy group 14 weeks after SCI. The myelin staining is light. The axon is located in the center of the concentric structure, and part of the grid structure has ruptured

MBP, GFAP, and NF and Nogo-A monoclonal SP antibody staining showed axons, myelin sheaths, and glial cells, respectively. Under an optical microscope at ×400, the results showed that: (1) the NF staining count was more increased at 14 weeks than at 6 weeks after SCI in the sham-operated group (Figs. 1 and 2) and in the 20-Gy irradiation group (Figs. 3 and 4) (*P* < 0.05), but there was no statistical significance at the two observation points in the 10-Gy irradiation group (Figs. 5 and 6); the NF staining count in the 10- and 20-Gy irradiation groups were increased significantly relative to the sham-operated group at 6 and 14 weeks after SCI (*P* < 0.05), but there were no differences between the two irradiation groups (Table 1). (2) The MBP staining count was increased significantly at 14 weeks as compared to 6 weeks after SCI in the sham-operated group (*P* < 0.05), and both of the two irradiation groups decreased significantly at 14 weeks as compared to 6 weeks after SCI. The MBP staining counts in irradiation groups were increased significantly at 6 weeks and decreased significantly at 14 weeks than those in sham-operated groups (*P* < 0.05) (Table 2); (3) The GFAP and Nogo-A staining count were increased more significantly at 14 weeks after SCI than at 6 weeks in each group (*P* < 0.05).

**Table 1:** NF numbers (‾*x* ± *s*) of sham SCI, 10 Gy irradiation, and 20 Gy irradiation rats

	6 weeks after SCI	14 weeks after SCI	*t*	*p*
0 Gy	8.4 ± 3.7 (*n* = 5)	31.5 ± 10.6 (*n =* 4)	−4.6	0.002
10 Gy	56.0 ± 23.9 (*n* = 6)*	77.2 ± 23.1 (*n* = 6)*	−1.56	0.150
20 Gy	30.5 ± 18.5 (*n* = 6)*,**	65.7 ± 27.4 (*n* = 6)*,**	−2.6	0.026
*F*	4.8	5.0		
*p*	0.025	0.025		

*P < 0.05 vs sham SCI (0-Gy group); **P > 0.05 vs 10-Gy group

**Table 2:** MBP numbers (‾*x* ± *s*) of sham SCI, 10 Gy irradiation, and 20 Gy irradiation rats

	6 weeks after SCI	14 weeks after SCI	*t*	*p*
0 Gy	134.0 ± 29.7 (*n* = 5)	251.5 ± 25.9 (*n* = 4)	−6.2	0.000
10 Gy	230.7 ± 7.1 (*n* = 6)*	160.8 ± 38.0 (*n* = 6)*	4.4	0.001
20 Gy	177.5 ± 25.2 (*n* = 6)*,**	140.0 ± 31.6 (*n* = 6)*,**	2.3	0.047
*F*	26.0	14.6		
*p*	0.000	0.000		

*P < 0.05 vs sham SCI (0-Gy group); **P > 0.05 vs 10-Gy group

## Discussion

In our earlier pre-experiments, the female rats had a lower infection rates and mortality than male rats. In order to ensure the consistency of the spinal cord injury model, we use the same sex rats and female SD rats were selected. The present study demonstrates that the anatomical recovery of injured area of the spinal cord made possibly by the x-ray treatment. However, the SCI rats exhibited no differences between the x-ray treatment group and sham-operated group at each point of observation (6 and 14 weeks after SCI) in the physiological observations, the BBB scores, and the SEP testing in our study. We suppose that the injuries of the rats may have been excessive, and subsequent histological examination also confirmed this conclusion. Neural axon is the basic function unit of spinal cord. In our study, the NF count of irradiation groups increased significantly than that of the sham groups, but the total quantity might not be enough, and all the experimental animals exhibit serious movement dysfunction so there are no differences among each group in the BBB scores and the SEP testing.

As we know, NF, MBP, and GFAP are classic histological indicators of the spinal cord. In our study, the NF staining count in the x-ray treatment group (including the 10- and 20-Gy group) was significantly increased relative to the sham-operated group at 6 and 14 weeks after SCI. No statistical significance was shown between the two radiation groups at each observation point. The results indicated that the irradiation treatment might promote the regeneration of the spinal cord central nervous system (CNS). In the sham-operated group, the NF count was increased notably at 14 weeks relative to that at 6 weeks, which showed that the spinal cord CNS has a self-repair mechanism after SCI. This process lasted at least 14 weeks. X-ray irradiation likely sped up the process and promoted the regeneration and rehabilitation of the spinal cord CNS to a certain extent. In this study, there was no significant difference between the 10- and the 20-Gy group. The experimental results were slightly different from those of Kalderon [1, 2, 4]. In the meantime, the neuromotor function and SEP inspection results showed no difference between sham-operated and x-ray treatment groups, which suggested that the quantity change did not match the qualitative change. Of course, the detection time was also not long enough [10, 11].

Myelin is a necessary structure of the CNS cells, and it might play a role in preventing the regeneration of nerves. Further research showed that the myelin-related protein Nogo-A could inhibit the regeneration of axons in vitro and in vivo [12, 13]. Our study showed that the MBP count at 14 weeks after SCI was increased significantly relative to that at 6 weeks in the sham-operated group. On the contrary, it was decreased in the x-ray treatment groups (both the 10- and 20-Gy groups) as time went on. Meanwhile, we found that the MBP count of the sham-operated group was significantly reduced relative to the x-ray treatment group at 6 weeks after SCI, and it increased at 14 weeks after SCI. This result showed that myelin experienced a decrease first and then increased after SCI in rats. This outcome also indicated that the injury and repair of the spinal cord were existed together, being injured first and then repairing it. The myelin count was significantly reduced in the irradiation group at week 14 after SCI, which showed that ionizing radiation also has long-term effects on the spinal CNS; the duration of this effect needs further research. In addition, we found that irradiation might promote the regeneration of the spinal cord in rats, which cannot be explained by the myelin theory only. We found that when the myelin count in the irradiation group was significantly increased at 6 weeks after SCI, Nogo-A did not concurrently increase. Possible inferences included the structure of the CNS tissue in early SCI, producing a sharp decay stage, while x-ray irradiation delayed the process and changed the tissue microenvironment, which was beneficial to the regeneration and rehabilitation of the CNS.

The GFAP monoclonal antibody could clearly indicate the normal and reactive astrocytes and the reticular structure of the spinal cord. The observed astrogliosis ultimately became an astrocyte glial scar. In our study, the glial scar gradually increased as time went on, while the irradiation group decreased relative to the sham-operated group during the same period (*P* > 0.05). This result showed that x-ray irradiation reduced the glial scar hyperplasia of CNS to a certain extent.

Nogo-A was highly expressed in the CNS oligodendrocytes and myelin. This result showed that the Nogo protein inhibits axonal regeneration in vivo and in vitro [12, 13]. However, in our study, the expression of the Nogo protein exhibited no significant difference between the irradiated group and the sham-operated group, which showed that x-ray irradiation plays a multifaceted and complex role in CNS regeneration.

## Conclusions

In the present study, we evaluated the action of x-ray irradiation on the regeneration of the spinal cord in rats using various methods objectively, and we proved that local x-irradiation could promote the regeneration of the spinal cord in a rat model. This mechanism of action needs to be further studied.
